# Diagnostic performance of a radiological Fagotti score assessed on diffusion-weighted magnetic resonance imaging for predicting tumor resectability in ovarian cancer patients: a feasibility study

**DOI:** 10.3389/fonc.2025.1680992

**Published:** 2025-10-27

**Authors:** Franziska Siegenthaler, Selma Zurbriggen, Corinne Wyss, Sara Imboden, Flurina Saner, Julian Wampfler, Lucine Christe, Wiebke Solass, Seline Hofer, Andreas Christe, Johannes T. Heverhagen, Michael D. Mueller, Verena C. Obmann

**Affiliations:** ^1^ Department of Obstetrics and Gynecology, Bern University Hospital and University of Bern, Bern, Switzerland; ^2^ Faculty of Medicine, University of Bern, Bern, Switzerland; ^3^ Department of Diagnostic, Interventional and Pediatric Radiology, Inselspital Bern, University Hospital, University of Bern, Bern, Switzerland; ^4^ Department of Medical Oncology, Bern University Hospital, Bern, Switzerland; ^5^ Institute of Tissue Medicine and Pathology, University of Bern, Bern, Switzerland; ^6^ Department of Radiology, Zuger Kantonsspital, Baar, Switzerland

**Keywords:** ovarian cancer, MRI, DWI (diffusion weighed images), Fagotti score, preoperative imaging

## Abstract

**Objective:**

In ovarian cancer, the correct selection of patients for cytoreductive surgery is of great importance. The aim of this study was to evaluate the diagnostic performance of the Fagotti score assessed on preoperative diffusion-weighted magnetic resonance imaging (DW-MRI) for predicting tumor resectability in patients with ovarian cancer.

**Methods:**

This retrospective cohort study included patients with ovarian cancer who underwent surgical treatment between 2014 and 2022 at University Hospital Bern, Switzerland. All patients had a preoperative MRI performed. The surgical and MRI Fagotti scores were assessed retrospectively, and follow-up data were available through standardized databases and follow-up controls.

**Results:**

A total of 52 imaging studies in 50 patients met the inclusion criteria. The majority of the patients presented with primary diagnosis (73.1%), serous histological subtype (75.0%), and advanced International Federation of Gynecology and Obstetrics (FIGO) stage (76.9%). The mean surgical Fagotti score was 3.4 (±3.24), while the mean MRI Fagotti score was 2.8 (±2.92). Consistency between the surgical and MRI assessments was 94.2% for stomach infiltration, 80.8% for superficial liver metastasis, and 80.8% for peritoneal, 80.8% for mesenteric, 78.8% for small bowel, 75.0% for omental, and 65.4% for diaphragmatic involvement. An MRI Fagotti score of <8 predicted a no residual disease (R0) resection with a sensitivity of 97.4%, a positive predictive value of 86.0%, and a negative predictive value of 83.3%. Patients with an MRI Fagotti score of <6 showed a significantly longer recurrence-free survival (log-rank, *p* = 0.020) and overall survival (log-rank, *p* = 0.043) compared to patients with an MRI Fagotti score of 6 or higher.

**Conclusion:**

According to the study results, the assessment of the Fagotti score on preoperative imaging with DW-MRI is feasible and useful in predicting tumor resectability in patients with ovarian cancer. Furthermore, an MRI Fagotti score higher than 6 is associated with worse oncological outcomes. However, further studies on a larger patient population are needed to confirm our results.

## Highlights

### What is already known on this topic?

The surgical Fagotti score is a laparoscopic predictive model for the selection of patients for cytoreductive surgery, whereby correct selection and R0 resection are of great prognostic importance. While the high diagnostic accuracy of diffusion-weighted magnetic resonance imaging (DW-MRI) in predicting suboptimal cytoreduction in both primary and recurrent disease has been previously demonstrated, a new preoperative MRI Fagotti score obtained from DW-MRI and its predictive value in assessing tumor resectability have not been analyzed yet.

### What this study adds

This is the first study to assess a preoperative MRI Fagotti score in DW-MRI to predict resectable disease in patients with ovarian cancer. The MRI Fagotti score was significantly correlated with the surgical Fagotti score, the surgical complexity score, and the oncologic outcome.

### How this study might affect research, practice, or policy

The MRI Fagotti score is important for preoperative assessment and surgical planning and could enable a more individualized patient treatment.

## Introduction

Ovarian cancer is the second most common cause of death due to gynecologic malignancy. The global incidence of ovarian cancer in 2020 was estimated to be 313,959, with 207,252 deaths (1). While localized stages have good 5-year survival of 95%, distant stages have a 5-year survival rate of 31% (2). Ovarian cancer is typically diagnosed at the advanced stages as there are no effective screening strategies and the early stages do not have obvious symptoms (3).

Currently, the gold standard for the treatment of advanced ovarian cancer is primary cytoreductive surgery if no residual disease (R0) can be achieved, followed by platinum-based chemotherapy. Neoadjuvant chemotherapy followed by interval cytoreductive surgery is a valid alternative (4). In patients with tumor recurrence, the DESKTOP III trial presented better overall survival in selected patients who underwent secondary cytoreductive surgery and chemotherapy compared with chemotherapy alone (5). The risk of recurrence in the advanced stages is high (70%–80%) and is often associated with post-surgical residues of the disease and resistance to chemotherapy (6).

In this context, imaging plays an essential role in the accurate selection of patients with resectable disease for cytoreductive surgery. Contrast-enhanced computed tomography (CT), magnetic resonance imaging (MRI), and positron emission tomography–computed tomography (PET-CT) are already used for the initial evaluation of patients. If complete cytoreductive surgery is in doubt based on the radiology report, laparoscopic assessment is a further option (4). Specialized MRI techniques such as diffusion-weighted imaging (DWI) have been proven to have better prediction not only of primary but also secondary suboptimal cytoreductive surgery than preoperative CT in primary disease and tumor recurrence (7, 8). DWI enables better diagnostic performance for peritoneal carcinomatosis (9, 10), which is often the case in advanced ovarian cancer (11). However, the clinical value of diffusion-weighted magnetic resonance imaging (DW-MRI) for the selection of patients for cytoreductive therapy in ovarian cancer has yet to be established.

Despite imaging advancements, diagnostic laparoscopy is the reference standard for evaluation of the tumor spread (12, 13). To improve patient selection, the Fagotti score was proposed in 2006 as a laparoscopy-based system for the prediction of the optimal chances of cytoreductive surgery. It evaluates seven anatomic areas (i.e., peritoneum, omentum, diaphragm, mesentery, bowel, stomach, and liver). A Fagotti score of 8 or higher indicates a specificity of 100% for suboptimal cytoreduction (14). However, there is no universal standard for the preoperative evaluation of peritoneal carcinomatosis or a common scoring system to correctly triage patients for cytoreductive surgery (4, 15). Hence, it is of great importance to determine preoperative evaluation of the tumor spread in order to correctly select patients for cytoreductive treatment.

The aim of this study was to evaluate whether the Fagotti score assessed on preoperative DW-MRI can predict tumor resectability in patients with ovarian cancer. Establishing a reliable MRI-based Fagotti score could improve preoperative patient selection, support surgical planning, and potentially reduce the need for invasive diagnostic laparoscopy.

## Methods

This retrospective cohort study included patients with ovarian cancer who underwent surgical treatment at the European Society of Gynaecological Oncology (ESGO)-certified cancer center at the University Hospital Bern, Switzerland, between April 2014 and November 2022. Patients with a primary diagnosis or a recurrence of ovarian cancer, aged ≥18 years, with preoperative MRI of the whole abdomen including the upper abdomen and the pelvis, who underwent surgical assessment of resectability, and who consented to follow-up were included. The local ethics committee in Bern, Switzerland, reviewed the study protocol (Req-2024-01003). All patients signed a written informed consent. In accordance with the journal guidelines, our data will be provided for independent analysis by a selected team by the Editorial Team for the purposes of additional data analysis or for the reproducibility of this study in other centers, if such is requested.

Clinicopathological and surgical data were retrieved from an electronic database. The Fagotti score includes peritoneal carcinomatosis, diaphragmatic involvement, mesenteric involvement, omental cake, small bowel involvement, stomach infiltration, and superficial liver metastasis, each contributing two points, resulting in a possible Fagotti score range of 0–14 (14). The surgical Fagotti score was either determined prospectively by the gynecologic oncologist at the time of surgery or, in patients without a previously documented Fagotti score, retrospectively by reviewing the surgical reports and the available video and photodocumentation. The gynecologic oncologist defining the surgical Fagotti score was blinded for the MRI Fagotti score and for patient survival.

The MRI Fagotti score was determined retrospectively by two trained radiologists with 5 and 12 years of gynecological imaging experience in consensus, who were blinded for the surgical Fagotti score, the resection status, and the patient survival, using T2- (T2W) and T1-weighted (T1W) non-contrast and contrast-enhanced and, when available, DW-MRI images. The images were read in the clinical used Picture Archiving and Communication System (PACS) (ID7; Sectra, Lindköping, Sweden). The MRI scans in our institution were performed on a 1.5-T (Aera) or a 3-T system (Skyra) from Siemens Healthineers (Erlangen, Germany). The dedicated MR protocol consisted of a T2W half-Fourier acquisition single-shot turbo spin echo sequence in axial and coronal orientations with a slice thickness of 5 and 6 mm, respectively. A 5-mm diffusion-weighted [echo-planar imaging (EPI) with fat suppression] and a 5-mm T1W [T1 volumetric interpolated breath-hold examination (VIBE) Dixon] sequence.

Follow-up data on recurrence and survival were available through standardized databases and follow-up controls. The clinical outcome parameters collected were overall survival and recurrence-free survival. Recurrence-free survival was defined as the time from the primary staging surgery to the first recurrence or death from any cause. Overall survival was calculated from the date of the primary staging surgery until death or until the date of the last follow-up.

Statistical analysis was performed using the Statistical Package for Social Sciences (IBM SPSS Statistic, version 28.0.1.1). Categorical variables were reported as frequencies and proportions, while continuous variables were reported as means and standard deviations. The sensitivity, the specificity, and the negative and positive predictive values to predict complete cytoreduction were calculated for the MRI Fagotti score. Formal comparisons were made using chi-square statistics (*χ* ^2^) or Fisher’s exact test for categorical variables and *t*-test or analysis of variance (ANOVA) for continuous variables. Survival analyses were performed using the Kaplan–Meier method and were compared using the log-rank test. Statistical significance was defined as a *p*-value below 0.05.

## Results

**Figure 1:**
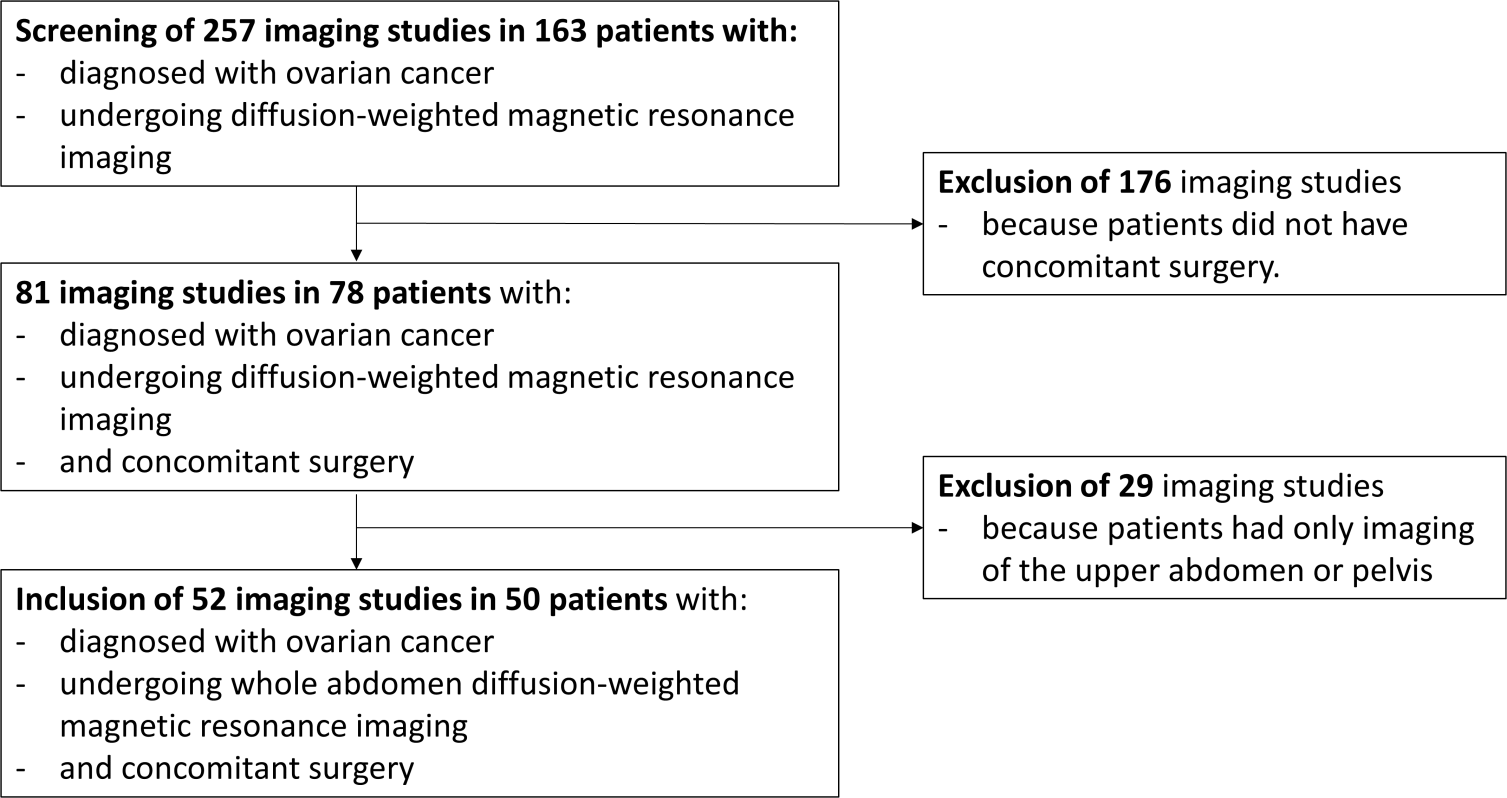
Flow diagram for patient inclusion.

In total, 257 imaging studies in 163 patients diagnosed with ovarian cancer were assessed for eligibility, with 52 imaging studies in 50 patients meeting the inclusion criteria (**Figure 1**). The main clinicopathological characteristics of the study cohort are described in **Table 1**. The majority of patients were included for primary diagnosis of ovarian cancer, while 26.9% presented with recurrent disease. The most common histological subtype, found in 75.0%, was serous carcinoma, and 65.4% of all patients presented with International Federation of Gynecology and Obstetrics (FIGO) stage III disease.

**Table 1:** Clinicopathological characteristics of the study cohort (*n* = 52 patients with ovarian cancer).

Whole study cohort (*n* = 52)	
Age at inclusion (years), mean ± SD	60.1 ± 14.9
BMI (kg/m^2^), mean ± SD	24.9 ± 5.6
Menopausal status, *n* (%)	
Premenopausal	10 (19.2)
Postmenopausal	34 (65.4)
Missing	8 (15.4)
CA-125 at inclusion (kU/L), mean ± SD	1,142.6 ± 3,887.1
Inclusion for, *n* (%)	
Primary diagnosis	38 (73.1)
Recurrence	14 (26.9)
Histological subtype, *n* (%)	
Serous	39 (75.0)
Mucinous	5 (9.6)
Endometrioid	2 (3.8)
Carcinosarcoma	2 (3.8)
Granulosa cell tumor	4 (7.7)
FIGO stage at inclusion, *n* (%)	
I	8 (15.4)
II	3 (5.8)
III	34 (65.4)
IV	6 (11.5)

Statistical analysis was performed using IBM SPSS Statistics, version 28.0.1.1. Results are presented as the mean ± SD for continuous variables and as absolute numbers with percentages for categorical variables.

*SD*, standard deviation; *BMI*, body mass index; *FIGO*, International Federation of Gynecology and Obstetrics.

All patients underwent preoperative MRI of the whole abdomen. DWI was available in 44 of 52 MR exams. MRI was performed on a 1.5-T (*n* = 29) or a 3-T scanner (*n* = 23).

Surgical data were available for all patients. At inclusion, 39 (75.0%) patients underwent a tumor debulking or staging surgery, while 13 (25%) patients underwent diagnostic laparoscopy alone. The reasons for not performing a tumor debulking surgery were unresectable disease in 10 patients, medical comorbidities in two patients, and unclear diagnosis in one patient. Of the 39 patients who underwent tumor debulking or staging surgery, a complete cytoreduction was achieved in 38 (97.4%). The mean operation time in these patients was 319 min (SD = 144 min), and the mean blood loss was 533 ml (SD = 442 ml). The mean surgical complexity score (16) was 5.1 (SD = 3.1). For all patients who underwent debulking or staging surgery, the surgical complexity was low in 11 (28.2%), intermediate in 18 (46.2%), and high in 10 (25.6%) patients. Four patients suffered intraoperative complications, including blood loss of more than 1 L in two patients, nerve injury in one patient, and a bladder lesion in one patient.

**Figure 2:**
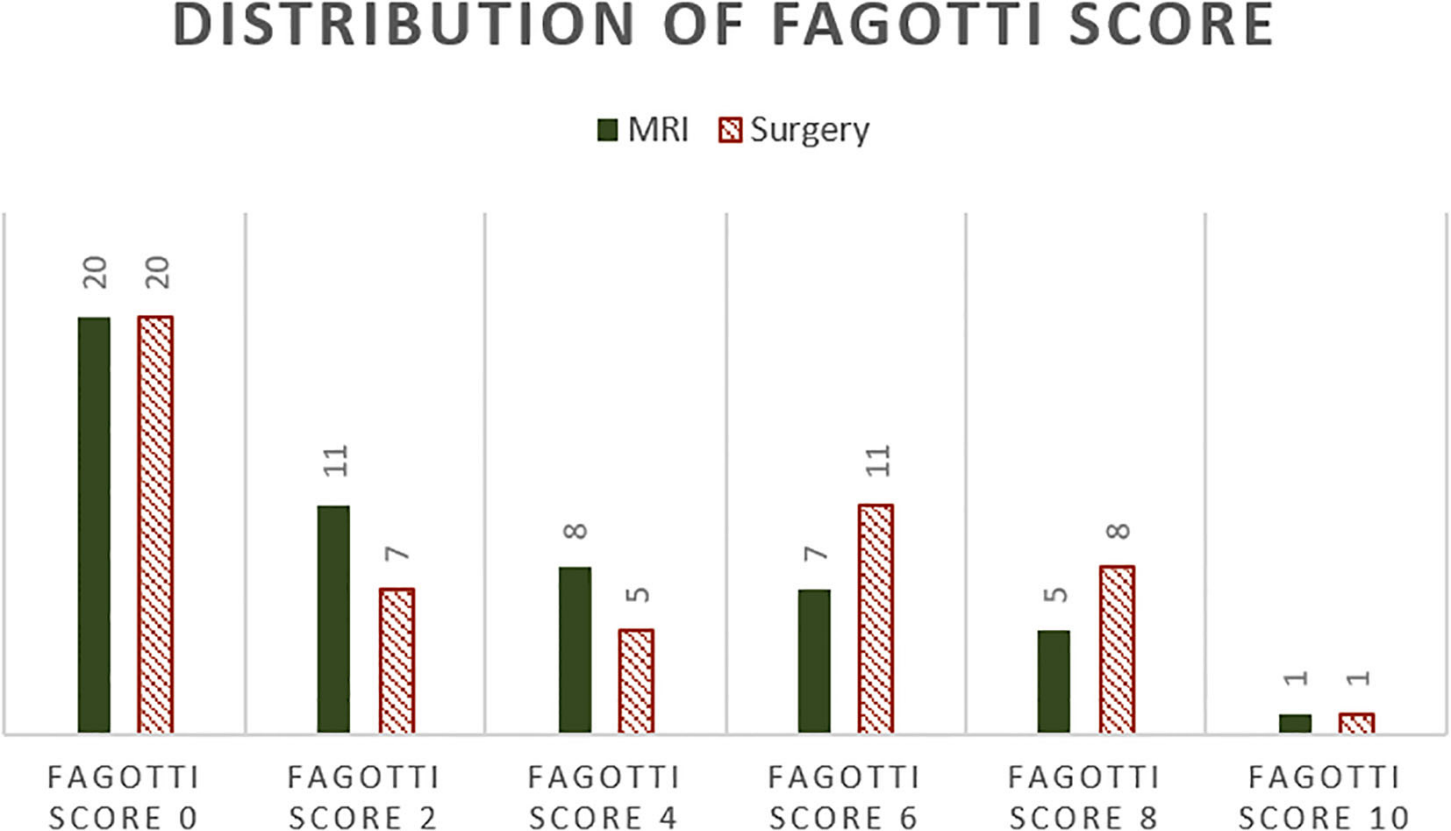
Distribution of the Fagotti scores assessed on MRI and during surgery.

**Figure 3:**
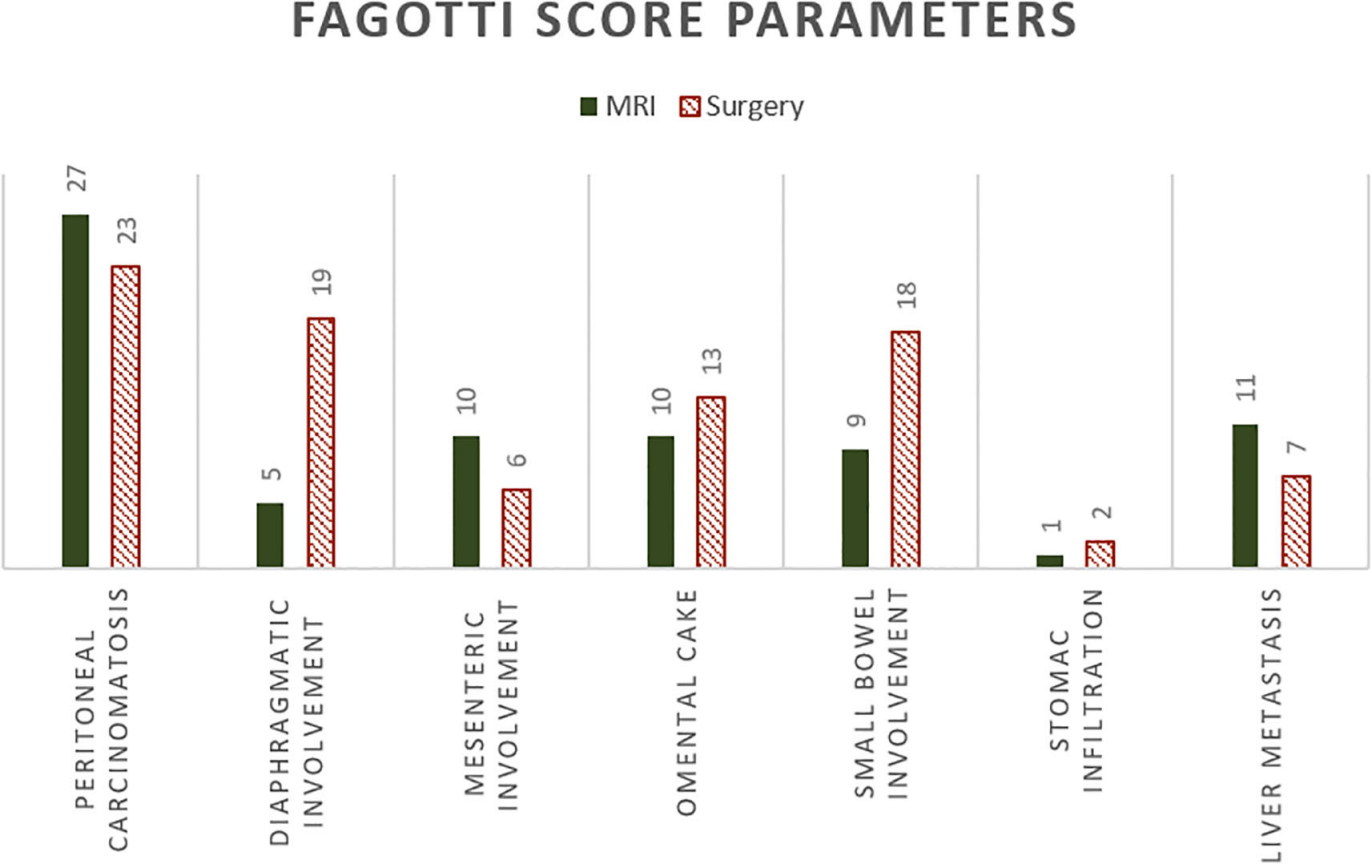
Detailed description of the Fagotti score parameters assessed on MRI and during surgery.

The surgical Fagotti score was assessed prospectively in 12 and retrospectively in 40 of the study participants. The mean surgical Fagotti score was 3.35 (SD = 3.24), while the mean MRI Fagotti score was 2.81 (SD = 2.92). The distribution of the Fagotti scores assessed during surgery and with MRI is displayed in **Figure 2**. The MRI and surgical Fagotti scores were congruent in 21 (40.4%) of the patients, MRI revealed a higher Fagotti score compared with surgery in 11 (21.2%) patients, and the surgical Fagotti score was higher compared with the MRI Fagotti score in 20 (38.5%) patients. **Figure 3** shows a detailed description of the Fagotti score parameters. Consistency between the surgical and MRI assessments was 80.8% for peritoneal carcinomatosis, 65.4% for diaphragmatic involvement, 80.8% for mesenteric involvement, 75.0% for omental cake, 78.8% for small bowel involvement, 94.2% for stomach infiltration, and 80.8% for liver metastasis. The surgical complexity score was significantly associated with the surgical (*p* < 0.001) and the MRI Fagotti scores (*p* = 0.010). The mean surgical complexity score was 4.5 (SD = 3.0) in patients with an MRI Fagotti score of <6 and was 7.8 (SD = 2.7) in those with an MRI Fagotti score of 6 or higher (*p* = 0.015). The MRI and the surgical Fagotti scores were significantly correlated with the surgical option of a complete cytoreduction (*p* = 0.003 and *p* = 0.016, respectively). An MRI Fagotti score of <8 predicted a complete cytoreduction with a sensitivity of 97.4%, a specificity of 45.5%, a positive predictive value of 86.0%, and a negative predictive value of 83.3% (see **Supplementary Table S2**).

**Figure 4:**
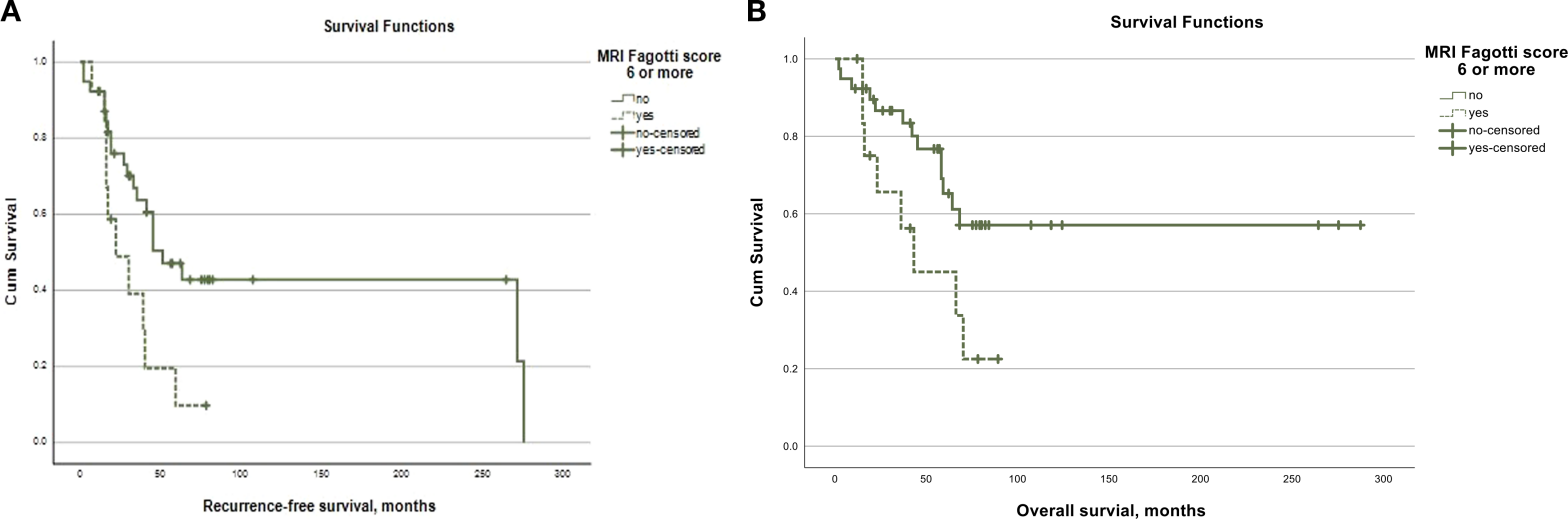
**(A)** Kaplan-Meier curve for recurrence-free survival according to the MRI Fagotti score. **(B)** Kaplan-Meier cureve for overall survival according to the MRI Fagotti score.

During a mean follow-up of 92.7 months (95%CI = 65.5–120.1), 27 (51.9%) patients recurred and 21 (40.4%) died. The recurrence-free survival was 133.4 months (95%CI = 77.3–149.6) for the whole study cohort. Patients with an MRI Fagotti score of <6 showed a significantly longer recurrence-free survival (134.0 months, 95%CI = 90.6–177.4) compared to those with a surgical Fagotti score of 6 or higher (32.2 months, 95%CI = 19.9–44.4; log-rank, *p* = 0.020) (**Figure 4A**). Similar associations were observed in terms of the overall survival: Patients with an MRI Fagotti score of <6 had a mean overall survival of 181.5 months (95%CI = 137.0–226.0) compared with 49.5 months (95%CI = 32.6–66.5) in those with an MRI Fagotti score of 6 or higher (log-rank, *p* = 0.043) (**Figure 4B**).

## Discussion

### Summary of the main results

This retrospective cohort study investigated the utility of the Fagotti score on preoperative imaging with DW-MRI in predicting tumor resectability in patients with ovarian cancer compared with the surgical Fagotti score obtained during laparoscopy. Consistency between the surgical and the MRI Fagotti scores was predominantly high for stomach infiltration, followed by liver metastasis and peritoneal, mesenteric, small bowel, and omental involvement, whereas diaphragmatic involvement presented with the worst consistency. The MRI and the surgical Fagotti scores significantly correlated with the selection of cytoreductive surgery and with the surgical complexity score. Furthermore, an MRI Fagotti score of <6 was associated with better recurrence-free survival and overall survival.

### Results in the context of the published literature

Complete surgical cytoreduction (R0) is one of the most important prognostic factors in patients with ovarian cancer (17). In our cohort, R0 resection was achieved in 97.4% of patients who underwent tumor debulking surgery, which is rather high when compared with other studies (9, 14, 18). The majority of patients had primary disease, serous carcinoma, and FIGO stage III cancer. The surgical complexity score was “intermediate” in half and was “high” in one-quarter of the study participants who underwent debulking or staging surgery. In a previous study, the disease distribution and burden were demonstrated to influence the complexity of the cytoreductive surgery required to achieve R0 resection (19). Moreover, the surgical complexity score was directly correlated with morbidity. Despite the increased risk of complications in more complex surgeries, it carried a survival benefit and showed that residual disease and surgical complexity held a prognostic significance (16). In our cohort, the surgical complexity score was significantly associated with the surgical and the MRI Fagotti scores. This validates that the MRI Fagotti score helps quantify the disease spread and eventually has an impact on the surgical complexity. It also highlights the comparability of the surgical and the MRI Fagotti scores. The mean surgical Fagotti score was higher than the mean MRI Fagotti score, which is in line with a recent study comparing the mean surgical peritoneal cancer index (PCI) with the mean DW-MRI PCI (20).

The high diagnostic accuracy of DW-MRI in predicting suboptimal cytoreduction in both primary and recurrent disease has been demonstrated before (7, 8). A previous study demonstrated a sensitivity of 94%, a specificity of 97.7%, and an accuracy of 95.7% for whole body DW-MRI, which are significantly higher for the prediction of suboptimal cytoreduction compared with CT (7). Particularly in certain regions of the peritoneum, lumbo-aortic lymph nodes, mesentery, pelvis, large bowel, and sigmoid–rectum, DW-MRI performed better than CT (21, 22). This underlines the importance of DW-MRI, especially as mesentery involvement, stomach infiltration, and liver metastasis are the least explorable areas during diagnostic laparoscopy (23). In this study, the consistency between the MRI and surgical assessments was highest at 94.2% for stomach infiltration, followed by 80.8% for superficial liver metastasis and 80.8% for peritoneal, 80.8% for mesenteric, 78.8% for small bowel, 75.0% for omental, and 65.4% for diaphragmatic involvement. A previous study that used DW-MRI and exploratory laparotomy showed the same accuracy for liver metastasis, stomach infiltration, peritoneal thickening, suprarenal para-aortic lymph nodes, and miliar visceral peritoneum. Only the diaphragm showed better accuracy in DW-MRI than during exploratory laparotomy for the prediction of suboptimal cytoreduction (24). In contrast, our cohort presented with more diaphragmatic involvement found during surgery compared with DW-MRI.

In this study, the MRI and surgical Fagotti scores were significantly correlated with the surgical option of a complete cytoreduction (*p* = 0.003 and *p* = 0.016, respectively). Similarly, studies have shown that, in serous high-grade ovarian cancer, the PCI derived from DW-MRI was correlated with the surgical PCI and was an independent predictor of complete cytoreduction (25, 26). This validates the benefit of preoperative evaluation of the peritoneal spread with DW-MRI, which supports our findings. Furthermore, a previously published study proposed a scoring model in a prospective evaluation of 34 patients. Nine different anatomic sites were each given one point. The authors achieved 91% accuracy for predicting incomplete tumor debulking using DW-MRI (24). In our study cohort, the best MRI Fagotti cutoff point was <8, with a sensitivity of 97.4%, a specificity 45.5%, a positive predictive value of 86%, and a negative predictive value of 83% for predicting complete resection. This corresponds with the performance of the surgical Fagotti score based on the tumor spread during diagnostic laparoscopy, demonstrating that a predictive index score of ≥8 would identify patients with suboptimal cytoreductive surgery with a sensitivity of 30% and a specificity of 100% (14).

To underline the clinical importance of DW-MRI for predicting resectable disease in ovarian cancer, we investigated the impact of the MRI Fagotti score on the oncological outcome. Patients with an MRI Fagotti score of <6 presented with significantly better recurrence-free survival and overall survival than those with an MRI Fagotti score of ≥6. This is in concordance with previously published studies that demonstrated worse overall and progression-free survival in patients who presented with unresectable disease during DW-MRI (8, 27). Furthermore, a recent study also demonstrated that the surgical Fagotti score is associated with progression-free and overall survival (28).

### Strengths and weaknesses

The main strength of this study is that it is the first study evaluating the Fagotti score on DW-MRI (MRI Fagotti) in patients with ovarian cancer. The study design included standardized imaging, blinded radiological scoring, and long-term clinical follow-up, which allowed assessment of both the surgical outcomes and oncological endpoints. This noninvasive approach has the potential to improve patient selection and reduce the need for diagnostic laparoscopy.

The limitations include the retrospective single-center design, the relatively small sample size, and the absence of external validation. The inter-observer variability between radiologists was not formally assessed, which should be addressed in future studies. Furthermore, the MRI protocols may differ across institutions. Therefore, prospective multicenter studies are needed to confirm the generalizability of our findings. There was no systematic comparison to other existing imaging predictors as CT scans were not available in all patients.

### Implications for practice and future research

It is of great importance to provide accurate preoperative evaluation and surgical planning as this impacts patient outcomes. This study demonstrates that the MRI Fagotti score can optimize patient selection for cytoreductive surgery with the benefit of preoperative assessment and better surgical planning, therefore allowing improved patient counseling. If these promising results are confirmed in larger cohorts and prospective trials, DW-MRI could serve as a valid addition to diagnostic laparoscopy, to determine whether complete resection is possible, while using the MRI Fagotti score to improve selection in patients with advanced cancer.

## Conclusion

In conclusion, the assessment of the Fagotti score on preoperative imaging with DW-MRI proved to be a valuable tool in predicting tumor resectability in patients with ovarian cancer. This is supported by the significant correlation observed between the MRI Fagotti score and the surgical Fagotti score, as well as their significant correlation with the surgical complexity score. Furthermore, an MRI Fagotti score of <6 was associated with better oncological outcomes. Therefore, using the MRI Fagotti score as an addition to diagnostic laparoscopy could lead to improved preoperative evaluation and better surgery planning, which enables more personalized counseling and treatment for women diagnosed with ovarian cancer. However, further prospective trials on a larger patient population are needed to confirm our findings.

## Data Availability

Many results from the Imaging Data and videos from the OR are stored on our systems and can only be exported on a pseudomized manner. However, in case of any doubt we can provide them on request. Requests to access these datasets should be directed to verena.obmann@zgks.ch.

## Supplementary material

Supplementary Table 1 Accuracy of MRI Fagotti score less than 6 to predict complete surgical cytoreduction. Excluding the three patients were a complete cytoreduction was abandonned due to other reasons.

Supplementary Table 2 Accuracy of MRI Fagotti score less than 8 to predict complete surgical cytoreduction. Excluding the three patients were a complete cytoreduction was abandonned due to other reasons.
