# Supplementary material for: Diagnostic performance of a radiological Fagotti score assessed on diffusion-weighted magnetic resonance imaging for predicting tumor resectability in ovarian cancer patients: a feasibility study
Source: Front Oncol. 2025 Oct 27;15:1680992. doi: 10.3389/fonc.2025.1680992 (PMC12597752; doi:10.3389/fonc.2025.1680992)
Supplement: Supplementary Table 1 — Accuracy of MRI Fagotti score less than 6 to predict complete surgical cytoreduction. Excluding the three patients were a complete cytoreduction was abandonned due to other reasons. [file Table1.pdf]

SUPPLEMENTARY MATERIALS

Table S1: Accuracy of MRI Fagotti score less than 6 to predict complete surgical cytoreduction

	Complete surgical cytoreduction	Complete surgical cytoreduction not possible due to unresectable disease	Total
Surgical Fagotti score < 6	32	5	37
Surgical Fagotti score 6 or more	6	6	12
Total	38	11	49*

*** *Excluding the three patients were a complete cytoreduction was abandonned due to other reasons***

Table S2: Accuracy of MRI Fagotti score less than 8 to predict complete surgical cytoreduction

	Complete cytoreduction possible	Complete cytoreduction not possible due to unresectable disease	Total
MRI Fagotti score < 8	37	6	43
MRI Fagotti score 8 or more	1	5	6
Total	38	11	49*

*** *Excluding the three patients were a complete cytoreduction was abandonned due to other reasons***
